# Pigmentary traits, sun exposure, and risk of non‐Hodgkin’s lymphoma/chronic lymphocytic leukemia: A study within the French E3N prospective cohort

**DOI:** 10.1002/cam4.3586

**Published:** 2020-11-21

**Authors:** Louis‐Marie Garcin, Amandine Gelot, Roselyn‐Rima Gomez, Gaëlle Gusto, Marie‐Christine Boutron‐Ruault, Marina Kvaskoff, Gianluca Severi, Caroline Besson

**Affiliations:** ^1^ Department of Medical Oncology Gustave Roussy Université Paris‐Saclay Villejuif France; ^2^ Hematology‐Oncology Department Centre Hospitalier de Versailles Le Chesnay France; ^3^ CESP Université Paris‐Saclay Univ. Paris‐Sud UVSQ INSERM Villejuif France; ^4^ Gustave Roussy Villejuif France; ^5^ Departement of Statistics, Computer Science and Applications (DISIA) University of Florence Florence Italy

**Keywords:** cohort study, keratinocyte cancer, nevi, non‐Hodgkin lymphoma

## Abstract

To investigate whether risk factors for keratinocyte carcinomas (KCs), namely pigmentary traits and sun exposure, are associated with risk of non‐Hodgkin's lymphomas (NHL) and chronic lymphocytic leukemia (CLL). E3N is a prospective cohort of French women aged 40–65 years at inclusion in 1990. Cancer data were collected at baseline and updated every 2–3 years. Hazard Ratios (HRs) and 95% confidence intervals (CIs) for associations between pigmentary traits and sun exposure, and risk of CLL/NHL were estimated using Cox models. With a median follow‐up of 24 years, 622 incident cases of CLL/NHL were ascertained among the 92,097 included women. The presence of nevi was associated with CLL/NHL risk: HR for “many or very many nevi” relative to “no nevi”: 1.56 [1.15; 2.11]. Such association with number of nevi appears to be mostly limited to risk of CLL: HR for “many or very many nevi”: 3.00 [1.38; 6.52]; versus 1.32 [0.94; 1.84] for NHL. Women whose skin was highly sensitive to sunburn also had a higher risk of CLL: HR = 1.96 [1.21; 3.18], while no increase in risk of NHL was observed. Skin or hair color, number of freckles, and average daily ultraviolet (UV) dose during spring and summer in location of residence at birth or at inclusion (kJ/m^2^) were not associated with CLL/NHL risk. Some pigmentary traits (presence of nevi and skin sensitivity), but not sun exposure, were associated with CLL/NHL. These observations suggest that CLL may share some constitutional risk factors with keratinocyte cancers.

## Graphical abstract

We investigated whether keratinocyte carcinomas risk factors were associated with Chronic lymphocytic leukemia (CLL) and non‐Hodgkin's lymphoma (NHL) risk. We report an association between risk of CLL/NHL and number of nevi. Our findings suggest a partially common genetic etiology of these tumors instead of shared environmental risk factors.

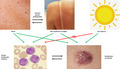

## 1 INTRODUCTION

Chronic lymphocytic leukemia (CLL) and non‐Hodgkin's lymphoma (NHL) are among the most common hematologic cancers. After a sharp increase between 1975 and 2000, their incidence has been stable since the beginning of the 2000s in France and other Northern countries. Main risk factors include exposure to pesticides, being a farmer, chronic viral infections with Human Immunodeficiency Virus (HIV) or hepatitis B or C viruses, and autoimmune diseases.^1^ Genome‐Wide Association Studies (GWAS) have identified several polymorphisms that modulate the risk of CLL and NHL.^2^, ^3^ Keratinocyte carcinomas (basal‐cell carcinoma (BCC) and squamous‐cell carcinoma (SCC)) are the most common cancers worldwide, and their incidence has been increasing for several decades. Several risk factors have been well established for these cancers, such as chronic exposure to ultraviolet (UV) radiation, a fair pigmentation, presence of nevi, and a family history of keratinocyte carcinomas.^4^, ^5^ The incidence of keratinocytes carcinomas (KCs) is also increased in patients with immune deficiency, especially after organ transplantation.^6^ Genetic predisposition factors have also been identified in several studies, including genome‐wide association studies.^7^, ^8^

Several epidemiological studies have reported associations between CLL/NHL and KCs, toward an increased risk of KCs following a diagnosis of CLL/NHL^9^, ^10^ or conversely.^11^ There is also a known epidemiological association between CLL/NHL and melanoma.^12^ Moreover, the risk factors for cutaneous carcinoma and melanoma are similar. KCs diagnosed in patients with previous lymphoid malignancies have been shown to be more aggressive and to have increased rates of recurrence, metastasis, and mortality.^9^, ^13^ Similarly, lymphoid malignancies diagnosed in patients with previous KCs are associated with higher rates of progression and recurrence.^14^ While the occurrence of a lymphoid malignancy as well as the administration of chemotherapy is associated with an immune deficiency likely to increase the risk of KCs, there is no obvious reason for the higher risk of CLL/NHL after prior diagnosis of KCs. Few studies explored potentially shared risk factors between KCs and CLL/NHL. Unlike KCs, for which the incidence increases with sun exposure, the incidence of CLL and NHL appears to be inversely associated with sun exposure,^15^ although some studies have published conflicting results.^16^, ^17^ We investigated whether KCs risk factors, namely pigmentary traits and sun exposure, were associated with CLL/NHL risk in the French E3 N prospective cohort.

## 2 METHODS

### 2.1 The E3N cohort

E3N (*Etude Epidémiologique auprès de femmes de l’Education Nationale*) is a prospective cohort study involving 98,995 women aged 40–65 years, living in metropolitan France at inclusion in 1990 and insured by the *Mutuelle générale de l’Éducation nationale*, a national health scheme covering workers from the National Education System and their families.^18^ Women were recruited after responding to a baseline self‐completed questionnaire on their lifestyle and medical background along with a signed informed consent. Tracking questionnaires were sent every two to three years since inclusion. The project obtained ethical support from the French National Commission for Data Protection and Privacy (*Commission Nationale Informatique et Libertés* (CNIL)).

### 2.2 Ascertainment of CLL/NHL cases

Medical data were collected at baseline and at each follow‐up questionnaire in the cohort. CLL/NHLs were categorized according to the classification of lymphoid neoplasms for epidemiologic research from the Pathology Working Group of the International Lymphoma Epidemiology Consortium (InterLymph).^19^ Occurrence of cancer was based on self‐reports from the questionnaires repeated every two to three years, next‐of‐kin spontaneous reports, and the national cause‐of‐death registry then validated through medical and pathology records. Cases that were ascertained only through death certificates were censored as non‐cases at the most recent completed questionnaire. So far, 82.5% of CLL/NHL cases were confirmed by pathology reports, medical records or death certificates.

### 2.3 Study population

CLL/NHL cases diagnosed during the 1990–2014 period were defined as incident cases. 2014 corresponded to the 11th questionnaire sent. To study the association between KC risk factors and CLL/NHL risk, we excluded from the original study population (n = 98,995) women with any cancer diagnosed before inclusion (with the exception of basal cell carcinoma and in situ colorectal tumor) (n = 4,848) and women lost to follow‐up after they answered the inclusion questionnaire (n = 2,050). Our final sample for analysis consisted of 92,097 women.

### 2.4 Assessment of exposure

In E3N, the baseline questionnaire inquired about self‐reported pigmentary characteristics including hair color (albinos, blond, red, chestnut, brown, and dark), skin complexion (albinos, very fair, fair, medium, dark, and very dark), number of nevi and number of freckles (none, few, many, and very many, for both), and skin sensitivity to sun exposure (low, moderate, and high). The questionnaire also inquired about places of birth and places of residence at baseline, which were linked to a database from the Joint Research Center of the European Commission (JRC) that contains average daily UV radiation doses (in kilojoules by square meter) in France^20^ to estimate average level of sun exposure.

### 2.5 Statistical analyses

We used Cox proportional hazards regression models stratified by birth generation with age as the time scale to compute hazard ratios (HRs) and 95% confidence intervals (CIs) for associations between pigmentary traits and CLL/NHL risk. Follow‐up time started at baseline in 1990 and ended at the earliest of the following times: date of the first diagnosis of cancer (with the exception of basal cell carcinoma and in situ colorectal tumor), date of last completed questionnaire until November 2014. Multivariable analyses of the main exposure variables were conducted when the p‐values of the univariate associations were below 0.20. We also ran a sensitivity analysis sub‐setting the CLL/NHL cases that were verified by medical records. The proportional hazard hypothesis was verified for all variables of interest using log‐log survivor plots. We have found no evidence of non‐proportional hazards. All tests of statistical significance were two‐sided, and significance was set at the 0.05 level. All analyses were performed using the SAS system, version 9.4 (SAS Institute).

## 3 RESULTS

During follow‐up (median time of 24 years), 622 incident cases of CLL/NHL were ascertained among the 92,097 E3N women included in the study. More precisely, there were 144 cases of CLL and 478 cases of NHL. Among the most commonly represented NHL subtypes, they were 108 diffuse large B‐cell lymphomas (DLBCL), 106 follicular lymphoma (FL), 57 marginal zone lymphoma (MZL), 30 Waldenstrom macroglobulinemia (WM), 12 T‐cell Lymphoma, and 11 mantle cell lymphoma (MCL). The mean age at diagnosis of CLL/NHL was 65.8 years (65.9 years for CLL and 65.6 years for NHL). (Table 1).

**TABLE 1:** Characteristics of study participants, E3N cohort 1990–2014 (N = 92,097)

		Case of NHL or CLL	
	All (N = 92,097)	Yes (N = 622)	No (N = 91,475)
Birth generation			
<1935	18,559 (20.1)	189 (30.4)	18,370 (20.1)
[1935; 1940]	17,226 (18.7)	142 (22.8)	17,084 (18.7)
[1940; 1945]	22,331 (24.3)	137 (22.0)	22,194 (24.3)
≥1945	33,981 (36.9)	154 (24.8)	33,827 (37.0)
Skin complexion			
Albinos, fair or very fair	52,866 (57.4)	345 (55.5)	52,521 (57.4)
Medium, dark or very dark	37,327 (40.5)	264 (42.4)	37,063 (40.5)
Missing value	1904 (2.1)	13 (2.1)	1891 (2.1)
Hair color			
Albinos, blond or red	10,787 (11.7)	58 (9.3)	10,729 (11.7)
Chestnut	53,882 (58.5)	378 (60.8)	53,504 (58.5)
Brown or dark	25,732 (27.9)	174 (28.0)	25,558 (27.9)
Missing value	1696 (1.9)	12 (1.9)	1684 (1.9)
Number of nevi			
Many or very many	47,807 (52.0)	328 (52.8)	47,479 (51.9)
A few	33,279 (36.1)	231 (37.1)	33,048 (36.1)
None	9066 (9.8)	48 (7.7)	9018 (9.9)
Missing value	1945 (2.1)	15 (2.4)	1930 (2.1)
Number of freckles			
Many or very many	31,242 (33.9)	214 (34.4)	31,028 (33.9)
A few	22,153 (24.1)	145 (23.3)	22,008 (24.1)
None	35,950 (39.0)	244 (39.2)	35,706 (39.0)
Missing value	2752 (3.0)	19 (3.1)	2733 (3.0)
Skin sensitivity to sunlight			
High	26,012 (28.2)	183 (29.4)	25,829 (28.2)
Moderate	43,105 (46.8)	275 (44.2)	42,830 (46.8)
Low	21,075 (22.9)	149 (24.0)	20,926 (22.9)
Missing value	1905 (2.1)	15 (2.4)	1890 (2.1)
Average daily UV dose during spring and summer in location of residence at birth (in kJ/m^2^)			
<2.36	20,036 (21.8)	124 (19.9)	19,912 (21.8)
[2.36; 2.48]	21,901 (23.8)	157 (25.2)	21,744 (23.8)
[2.48; 2.69]	20,154 (21.9)	158 (25.4)	19,996 (21.9)
≥2.69	22,365 (24.3)	134 (21.6)	22,231 (24.2)
Missing value	7641 (8.2)	49 (7.9)	7592 (8.3)
Average daily UV dose during spring and summer in location of residence at birth (in kJ/m^2^) (N = 84,456)	2.5 (0.2)	2.5 (0.2)	2.5 (0.2)
Average daily UV dose during spring and summer in location of residence at inclusion (in kJ/m^2^)			
<2.36	20,128 (21.9)	133 (21.4)	19,995 (21.9)
[2.36; 2.48]	25,685 (27.9)	184 (29.6)	25,501 (27.9)
[2.48; 2.70]	22,918 (24.9)	156 (25.1)	22,762 (24.9)
≥2.70	23,339 (25.3)	149 (24.0)	23,190 (25.4)
Missing value	27 (<0.1)	0 (<0.1)	27 (<0.1)
Average daily UV dose during spring and summer in location of residence at inclusion (in kJ/m^2^) (N = 92,070)	2.6 (0.3)	2.5 (0.3)	2.6 (0.3)

N(%) and *p*‐value of Khi‐2 test for categorical variables. Mean (STD) and logistic regression test for continuous variables.

Abbreviations: kJ/m^2^, kilojoules by square meter; UV; ultraviolet.

The presence of nevi was associated with CLL/NHL risk: HR for nevi frequency compared to no nevi was 1.42 [1.04; 1.94] for few and 1.56 [1.15; 2.11] for many or very many nevi, *p*‐trend = 0.007. (Table 2) Associations with nevi frequency were mostly observed with CLL for which a linear trend was observed (few: HR = 1.86 [0.84; 4.15]; many or very many: HR = 3.00 [1.38; 6.52]; vs. none; *p*‐trend = 0.0003). Associations with NHL were lower and not statistically significant (few: HR = 1.34 [0.96; 1.88]; many or very many: HR = 1.32 [0.94; 1.84]; vs. none; *p*‐trend = 0.26). We also studied this association specifically in the DLBCL and FL subgroups. There was no association between number of nevi and DLBCL or FL. (Table S1). We found no association between a high skin sensitivity to sunlight and the risk of CLL/NHL overall (HR = 1.08 [0.87; 1.35]). However, women whose skin was highly sensitive to sunburn were at a higher risk of CLL (HR = 1.96 [1.21; 3.18]), while no increase in NHL risk was observed (HR = 0.92 [0.72; 1.17]). Skin or hair color, number of freckles, and average daily ultraviolet (UV) dose during spring and summer in location of residence at birth or at inclusion (kJ/m^2^) were not associated with CLL/NHL risk. There was some non‐statistically significant inverse association between levels of residential sun exposure at baseline and CLL/NHL risk: (HR = 1.06 [0.85; 1.33] for the second quartile of exposure relative to the first; HR = 0.98 [0.78; 1.24] for the third quartile; HR = 0.90 [0.72; 1.14] for the fourth quartile, *p*‐ trend = 0.42). The sensitivity analysis, restricted to the 82.5% of cases confirmed by medical records, did not modify the results. (Table S2).

**TABLE 2:** Association between KC risk factors and risk of CLL/NHL; E3N cohort 1990–2014 (n = 92,097)*

	CLL/NHL		NHL		CLL	
	Number of cases (%) N = 622	HR [95% CI]	Number of cases (%) N = 478	HR [95% CI]	Number of cases (%) N = 144	HR [95% CI]
Skin sensitivity to sunlight						
Low	149 (23.95)	Reference	125 (26.15)	Reference	24 (16.67)	Reference
Moderate	275 (44.21)	0.95 [0.77; 1.16]	215 (44.98)	0.88 [0.70; 1.09]	60 (41.67)	1.30 [0.81; 2.10]
High	183 (29.42)	1.08 [0.87; 1.35]	131 (27.41)	0.92 [0.72; 1.17]	52 (36.11)	1.96 [1.21; 3.18]
*p*‐trend		0.41		0.53		**0.004**
Number of nevi						
None	48 (7.72)	Reference	41 (8.58)	Reference	7 (4.86)	Reference
A few	231 (37.14)	1.42 [1.04; 1.94]	188 (39.33)	1.34 [0.95; 1.88]	43 (29.86)	1.86 [0.84; 4.15]
Many or very many	328 (52.73)	1.56 [1.15; 2.11]	241 (50.42)	1.32 [0.94; 1.84]	87 (60.42)	3.00 [1.38; 6.52]
*p*‐trend		**0.007**		0.26		**0.0003**
Skin complexion						
Albinos, fair or very fair	345 (55.47)	Reference	263 (55.02)	Reference	82 (56.94)	Reference
Medium, dark or very dark	264 (42.44)	1.05 [0.89; 1.23]	209 (43.72)	1.09 [0.91; 1.30]	55 (38.19)	0.91 [0.65; 1.28]
*p*‐trend		0.59		0.36		0.59
Hair color						
Albinos, blond or red	58 (9.32)	Reference	47 (9.83)	Reference	11 (7.64)	Reference
Chestnut	378 (60.77)	1.28 [0.97; 1.68]	288 (60.25)	1.20 [0.88; 1.64]	90 (62.50)	1.59 [0.85; 2.98]
Brown or dark	174 (27.97)	1.24 [0.92; 1.67]	138 (28.87)	1.21 [0.87; 1.69]	36 (25.00)	1.35 [0.69; 2.66]
*p*‐trend		0.34		0.36		0.75
Number of freckles						
None	244 (39.23)	Reference	194 (40.59)	Reference	50 (34.72)	Reference
A few	145 (23.31)	0.98 [0.80; 1.20]	110 (23.01)	0.93 [0.74; 1.18]	35 (24.31)	1.16 [0.75; 1.78]
Many or very many	214 (34.41)	1.05 [0.87; 1.26]	163 (34.10)	1.00 [0.81; 1.23]	44 (30.56)	1.23 [0.83; 1.81]
*p*‐trend		0.65		0.96		0.30
Average daily UV dose during spring and summer in location of residence at birth (kJ/m^2^)						
Quartile 1: <2.36	124 (19.94)	Reference	89 (18.62)	Reference	35 (24.31)	Reference
Quartile 2: 2.36–2.48	157 (25.24)	1.15 [0.91; 1.45]	119 (24.90)	1.21 [0.92; 1.59]	38 (26.39)	0.99 [0.62; 1.56]
Quartile 3: 2.48–2.69	158 (25.40)	1.23 [0.97; 1.56]	127 (26.57)	1.38 [1.05; 1.81]	31 (21.53)	0.86 [0.53; 1.39]
Quartile 4: ≥2.69	134 (21.54)	0.95 [0.74; 1.21]	101 (21.13)	1.00 [0.75; 1.33]	33 (22.92)	0.83 [0.52; 1.34]
*p*‐trend		0.91		0.67		0.33
Average daily UV dose during spring and summer in location of residence at inclusion (kJ/m^2^)						
Quartile 1: <2.36	133 (21.38)	Reference	103 (21.55)	Reference	30 (20.83)	Reference
Quartile 2: 2.36 ‐ 2.48	184 (29.58)	1.06 [0.85; 1.33]	139 (29.08)	1.04 [0.80; 1.34]	45 (31.25)	1.15 [0.73; 1.83]
Quartile 3: 2.48 ‐ 2.69	156 (25.08)	0.98 [0.78; 1.24]	121 (25.31)	0.98 [0.76; 1.28]	35 (24.31)	0.97 [0.60; 1.58]
Quartile 4: ≥2.69	149 (23.95)	0.90 [0.72; 1.14]	115 (24.06)	0.90 [0.69; 1.18]	34 (23.61)	0.91 [0.56; 1.48]
*p*‐trend		0.42		0.64		0.41

Adjusted for age (as the time‐scale), stratified by birth generation (< 1930, [1930; 1935], [1935; 1940], [1940; 1945], ≥1945). Significance, indicated in bold values, was set at the 0.05 level.

Abbreviations: CI, Confidence Interval; HR, Hazard‐Ratio; kJ/m^2^, kilojoules by square meter; UV, ultraviolet.

* For cases of CLL or NHL, totals do not add up because the category of missing values for each factor was not included in the table. There were 15 (2.41%) for skin sensitivity to sunlight; 15 (2.41%) for number of nevi; 13 (2.09%) for skin complexion; 12 (1.93%) for hair color; 19 (3.05%) for number of freckles; 49 (7.88%) for the average UV dose during spring and summer in location of residence at birth and no cases with missing values for the average UV dose during spring and summer in location of residence at inclusion.

Moreover, a multivariable analysis was performed for the CLL category and included "number of nevi" and "skin sensitivity to sunlight." The associations remained highly significant. (Table 3).

**TABLE 3:** Association between “number of nevi,” “skin sensibility to sunlight” and risk of CLL; E3N cohort 1990–2014 (n = 92,097)—MULTIVARIABLE ANALYSIS

	CLL Number of cases (%) N = 144	HR [95% CI]
Skin sensitivity to sunlight		
Low	24 (16.67)	Reference
Moderate	60 (41.67)	1.27 [0.79; 2.04]
High	52 (36.11)	1.91 [1.18; 3.11]
*p*‐trend		**0.005**
Number of nevi		
None	7 (4.86)	Reference
A few	43 (29.86)	1.90 [0.85; 4.23]
Many or very many	87 (60.42)	3.00 [1.38; 6.50]
*p*‐trend		**0.0004**

Adjusted for age (as the time‐scale), stratified by birth generation (<1930, [1930; 1935], [1935; 1940], [1940; 1945], ≥1945). Significance, indicated in bold values, was set at the 0.05 level.

Abbreviations: CI, Confidence Interval; HR, Hazard‐Ratio.

* For cases of CLL, totals do not add up because the category of missing values for each factor was not included in the table. There were 8 (5.56%) for skin sensitivity to sunlight; 7 (4.86%) for number of nevi.

## 4 DISCUSSION

This study investigated whether risk factors for keratinocyte carcinomas (KCs), namely pigmentary traits and sun exposure, were associated with risk of CLL/NHL. Nevi frequency and skin sensitivity were associated with CLL risk. In contrast, there was a trend toward an inverse association between UV exposure and risk of CLL/NHL.

The association between number of nevi and risk of CLL/NHL could be explained by common constitutional risk factors. Even if childhood sun exposure plays a role in nevus acquisition,^21^, ^22^ twin studies have congruently shown genetic heritability of number of nevi with an estimated 40%–80% of the variance in nevus counts being assignable to genetic explanations.^23^ Our findings, therefore, support the hypothesis of a genetic mechanism contributing to the association between nevi and CLL/NHL. Interestingly, whole‐genome studies suggest the contribution of genetic factors common to both types of cancer.^24^ A previous study reported associations between several genetic variants associated with the risk of KCs and the risk of CLL, and conversely.^25^ In particular, the strongest associations were observed within the IRF4 gene locus (6p25.3), whose polymorphisms have also been associated with skin color in whole‐genome studies,^26^ and with CLL risk. These results raise the hypothesis of a pleiotropic effect of these genes on the pathogenesis of KCs and lymphoid malignancies. Similarly, there are probably common signaling pathways between CLL/NHL and melanoma. The *bcl*‐*2* oncogene, an inhibitor of apoptosis, is overexpressed in many lymphomas as well as in melanomas.^27^ The gene p16 may also potentially be involved in the occurrence of these malignancies since it inhibits the cyclin‐dependent kinase CDK4 (CD4 K and CDKN2A being known melanoma susceptibility genes) and since some mutations of p16 were reported in different types of lymphoma.^12^

Our study had several strengths, particularly its prospective design, large sample size, over 25 years of follow‐up, detailed assessment of risk factors for KCs through a baseline questionnaire collecting information on both pigmentary traits (number of nevi and freckles, skin and hair color, and skin sensitivity to sun exposure), and environmental factors (average level of sun exposure). A limitation of the study includes self‐declaration of number of nevi using a qualitative grid. Last, there was a lack of data on recreational UV exposure and on CLL/NHL risk factors (e.g., exposure to solvents or pesticides, chronic viral infection history, previous history of immune deficiency). In conclusion, we report an association between nevi frequency and CLL/NHL risk, suggesting a partly common genetic etiology of these tumors. Future research should investigate common pathophysiological pathways that could promote the development of both skin carcinoma and CLL/NHL.

## CONFLICTS OF INTEREST

The authors have no conflict of interest to declare. Data could be available upon request to the corresponding author.

## AUTHOR CONTRIBUTION

Louis‐Marie Garcin participated in the study conception and in the interpretation of the findings. He wrote the paper with Caroline Besson. Amandine Gelot, Roselyn‐Rima Gomez, and Gaelle Gusto performed the statistical analysis. Marina Kvaskoff, Marie‐Christine Boutron‐Ruault, and Caroline Besson conceived and designed the study. Gianluca Severi supervised the analysis and reviewed the paper.

## Supporting information

Table S1

Table S2

## References

[1] Morton LM , Slager SL , Cerhan JR , et al. Etiologic heterogeneity among non‐Hodgkin lymphoma subtypes: the InterLymph Non‐Hodgkin Lymphoma Subtypes Project. J Natl Cancer Inst Monographs. 2014;2014(48):130–144. 10.1093/jncimonographs/lgu013 PMC4155467 25174034

[2] Cerhan JR , Berndt SI , Vijai J , et al. Genome‐wide association study identifies multiple susceptibility loci for diffuse large B cell lymphoma. Nat Genet. 2014;46(11):1233–1238. 25261932 10.1038/ng.3105 PMC4213349

[3] Skibola CF , Berndt SI , Vijai J , et al. Genome‐wide association study identifies five susceptibility loci for follicular lymphoma outside the HLA region. Am J Hum Genet. 2014;95(4):462–471. 25279986 10.1016/j.ajhg.2014.09.004 PMC4185120

[4] Khalesi M , Whiteman DC , Tran B , Kimlin MG , Olsen CM , Neale RE . A meta‐analysis of pigmentary characteristics, sun sensitivity, freckling and melanocytic nevi and risk of basal cell carcinoma of the skin. Cancer Epidemiol. 2013;37(5):534–543. 23849507 10.1016/j.canep.2013.05.008

[5] Asgari MM , Warton EM , Whittemore AS . Family history of skin cancer is associated with increased risk of cutaneous squamous cell carcinoma. Dermatol Surg. 2015;41(4):481–486. 25760557 10.1097/DSS.0000000000000292 PMC5758040

[6] Iannacone MR , Sinnya S , Pandeya N , et al. Prevalence of skin cancer and related skin tumors in high‐risk kidney and liver transplant recipients in Queensland. Australia. J Invest Dermatol. 2016;136(7):1382–1386. 26968258 10.1016/j.jid.2016.02.804

[7] Chahal HS , Lin Y , Ransohoff KJ , et al. Genome‐wide association study identifies novel susceptibility loci for cutaneous squamous cell carcinoma. Nat Commun. 2016;18(7):12048. 10.1038/ncomms12048 PMC4960294 27424798

[8] Chahal HS , Wu W , Ransohoff KJ , et al. Genome‐wide association study identifies 14 novel risk alleles associated with basal cell carcinoma. Nat Commun. 2016;19(7):12510. 10.1038/ncomms12510 PMC4992160 27539887

[9] Brewer JD , Habermann TM , Shanafelt TD . Lymphoma‐associated skin cancer: incidence, natural history, and clinical management. Int J Dermatol. 2014;53(3):267–274. 24320558 10.1111/ijd.12208

[10] Engels EA , Parsons R , Besson C , et al. Comprehensive evaluation of medical conditions associated with risk of non‐Hodgkin Lymphoma using Medicare Claims (“MedWAS”). Cancer Epidemiol Biomarkers Prev. 2016;25(7):1105–1113. 27197296 10.1158/1055-9965.EPI-16-0212 PMC4930732

[11] Ransohoff KJ , Stefanick ML , Li S , et al. Association of nonmelanoma skin cancer with second noncutaneous malignancy in the Women’s Health Initiative. Br J Dermatol. 2017;176(2):512–516. 27229371 10.1111/bjd.14766

[12] McKenna DB , Stockton D , Brewster DH , Doherty VR . Evidence for an association between cutaneous malignant melanoma and lymphoid malignancy: a population‐based retrospective cohort study in Scotland. Br J Cancer. 2003;88(1):74–78. 12556962 10.1038/sj.bjc.6600692 PMC2376790

[13] Falchi L , Vitale C , Keating MJ , et al. Incidence and prognostic impact of other cancers in a population of long‐term survivors of chronic lymphocytic leukemia. Ann Oncol. 2016;27(6):1100–1106. 26912560 10.1093/annonc/mdw072 PMC4880062

[14] Toro JR , Blake PW , Björkholm M , Kristinsson SY , Wang Z , Landgren O . Prior history of non‐melanoma skin cancer is associated with increased mortality in patients with chronic lymphocytic leukemia. Haematologica. 2009;94(10):1460–1464. 19794092 10.3324/haematol.2008.004721 PMC2754966

[15] Bassig BA , Lan Q , Rothman N , Zhang Y , Zheng T . Current understanding of lifestyle and environmental factors and risk of non‐hodgkin lymphoma: an epidemiological update. J Cancer Epidemiol. 2012;2012:978930. 23008714 10.1155/2012/978930 PMC3447374

[16] Chang ET , Canchola AJ , Cockburn M , et al. Adulthood residential ultraviolet radiation, sun sensitivity, dietary vitamin D, and risk of lymphoid malignancies in the California Teachers Study. Blood. 2011;118(6):1591–1599. 21622649 10.1182/blood-2011-02-336065 PMC3156047

[17] Slager SL , Benavente Y , Blair A , et al. Medical history, lifestyle, family history, and occupational risk factors for chronic lymphocytic leukemia/small lymphocytic lymphoma: the InterLymph Non‐Hodgkin Lymphoma Subtypes Project. J Natl Cancer Inst Monographs. 2014;2014(48):41–51. 10.1093/jncimonographs/lgu001 PMC4155456 25174025

[18] Clavel‐Chapelon F . E3N Study Group. Cohort profile: The French E3N cohort study. Int J Epidemiol. 2015;44(3):801–809. 25212479 10.1093/ije/dyu184

[19] Morton LM , Turner JJ , Cerhan JR , et al. Proposed classification of lymphoid neoplasms for epidemiologic research from the Pathology Working Group of the International Lymphoma Epidemiology Consortium (InterLymph). Blood. 2007;110(2):695–708. 17389762 10.1182/blood-2006-11-051672 PMC1924473

[20] Verdebout J . A European satellite‐derived UV climatology available for impact studies. Radiat Prot Dosimetry. 2004;111(4):407–411. 15550711 10.1093/rpd/nch063

[21] Dodd AT , Morelli J , Mokrohisky ST , Asdigian N , Byers TE , Crane LA . Melanocytic nevi and sun exposure in a cohort of colorado children: anatomic distribution and site‐specific sunburn. Cancer Epidemiol Biomarkers Prev. 2007;16(10):2136–2143. 17932362 10.1158/1055-9965.EPI-07-0453 PMC2997330

[22] Whiteman DC , Brown RM , Purdie DM , Hughes M‐C . Melanocytic nevi in very young children: the role of phenotype, sun exposure, and sun protection. J Am Acad Dermatol. 2005;52(1):40–47. 15627079 10.1016/j.jaad.2004.07.053

[23] Wachsmuth RC , Turner F , Barrett JH , et al. The effect of sun exposure in determining nevus density in UK adolescent twins. J Invest Dermatol. 2005;124(1):56–62. 15654953 10.1111/j.0022-202X.2004.23548.x

[24] Li L , Ruau DJ , Patel CJ , et al. Disease risk factors identified through shared genetic architecture and electronic medical records. Sci Transl Med. 2014;6(234):234ra57. 10.1126/scitranslmed.3007191 PMC4323098 24786325

[25] Besson C , Moore A , Wu W , et al. Common genetic polymorphisms contribute to the association between non‐melanoma skin cancer and chronic lymphocytic Leukemia. Blood. 2017;130(Suppl 1):1454.

[26] Liu F , Visser M , Duffy DL , et al. Genetics of skin color variation in Europeans: genome‐wide association studies with functional follow‐up. Hum Genet. 2015;134(8):823–835. 25963972 10.1007/s00439-015-1559-0 PMC4495261

[27] McIntosh BC , Ariyan S , Esche G , Zelterman D , Narayan D . Metachronous primary melanoma and lymphoma. Ann Plast Surg. 2010;64(2):229–232. 20098111 10.1097/SAP.0b013e3181a13dbf

