# Supplementary material for: Pigmentary traits, sun exposure, and risk of non‐Hodgkin’s lymphoma/chronic lymphocytic leukemia: A study within the French E3N prospective cohort
Source: Cancer Med. 2020 Nov 21;10(1):297–304. doi: 10.1002/cam4.3586 (PMC7826467; doi:10.1002/cam4.3586)
Supplement: Supplementary file 2 — Table S2 [file CAM4-10-297-s002.doc]

**Supplementary Table S2: Association between KC risk factors and risk of CLL/NHL; E3N cohort 1990-2014 (n= 92,097) – SENSITIVITY ANALYSIS among cases confirmed by medical records**

|  | **CLL/NHL** | | **NHL** | | **CLL** | |
| --- | --- | --- | --- | --- | --- | --- |
|  | **Number of cases (%) N=513** | **HR [95% CI]** | **Number of cases (%)  N=378** | **HR [95% CI]** | **Number of cases (%) N=135** | **HR [95% CI]** |
| Skin sensitivity to sunlight |  |  |  |  |  |  |
| Low | 109 (21.25) | Reference | 88 (23.28) | Reference | 21 (15.56) | Reference |
| Moderate | 233 (45.42) | 1.09 [0.86; 1.36] | 176 (46.56) | 1.01 [0.78; 1.30] | 57 (42.22) | 1.42 [0.86; 2.34] |
| High | 157 (30.60) | 1.25 [0.98; 1.60] | 108 (28.57) | 1.05 [0.79; 1.40] | 49 (36.30) | 2.11 [1.26; 3.52] |
| P-trend |  | 0.06 |  | 0.69 |  | **0.003** |
| Number of nevi |  |  |  |  |  |  |
| None | 31 (6.04) | Reference | 25 (6.61) | Reference | 6 (4.44) | Reference |
| A few | 189 (36.84) | 1.77 [1.21; 2.60] | 148 (39.15) | 1.70 [1.11; 2.59] | 41 (30.37) | 2.08 [0.88; 4.89] |
| Many or very many | 279 (54.39) | 2.00 [1.37; 2.90] | 198 (52.38) | 1.71 [1.12; 2.59] | 81 (60.00) | 3.26 [1.42; 7.50] |
| P-trend |  | **0.0006** |  | 0.06 |  | **0.0005** |
| Skin complexion |  |  |  |  |  |  |
| Albinos, fair or very fair | 288 (56.14) | Reference | 211 (55.82) | Reference | 77 (57.04) | Reference |
| Medium, dark or very dark | 213 (41.52) | 1.02 [0.85; 1.21] | 162 (42.86) | 1.06 [0.86; 1.30] | 51 (37.78) | 0.90 [0.63; 1.28] |
| P-trend |  | 0.87 |  | 0.59 |  | 0.55 |
| Hair color |  |  |  |  |  |  |
| Albinos, blond or red | 46 (8.97) | Reference | 35 (9.26) | Reference | 11 (8.15) | Reference |
| Chestnut | 315 (61.40) | 1.34 [0.99; 1.83] | 231 (61.11) | 1.30 [0.91; 1.85] | 84 (62.22) | 1.49 [0.79; 2.79] |
| Brown or dark | 141 (27.49) | 1.27 [0.91; 1.77] | 108 (28.57) | 1.28 [0.87; 1.87] | 33 (24.44) | 1.24 [0.63; 2.46] |
| P-trend |  | 0.41 |  | 0.37 |  | 0.91 |
| Number of freckles |  |  |  |  |  |  |
| None | 201 (39.18) | Reference | 152 (40.21) | Reference | 49 (36.30) | Reference |
| A few | 115 (22.42) | 0.94 [0.74; 1.18] | 84 (22.22) | 0.90 [0.69; 1.18] | 31 (22.96) | 1.05 [0.67; 1.64] |
| Many or very many | 179 (34.89) | 1.05 [0.86; 1.29] | 132 (34.92) | 1.02 [0.81; 1.29] | 47 (34.81) | 1.15 [0.77; 1.72] |
| P-trend |  | 0.62 |  | 0.88 |  | 0.48 |
| Average daily UV dose during spring and summer in country of residence at birth (in kJ/m²) |  |  |  |  |  |  |
| < 2.36 | 106 (20.66) | Reference | 71 (18.78) | Reference | 35 (25.93) | Reference |
| [2.36 ; 2.48[ | 122 (23.78) | 1.05 [0.81; 1.36] | 90 (23.81) | 1.15 [0.84; 1.57] | 32 (23.70) | 0.83 [0.51; 1.34] |
| [2.48 ; 2.69[ | 136 (26.51) | 1.24 [0.96; 1.60] | 106 (28.04) | 1.45 [1.07; 1.95] | 30 (22.22) | 0.83 [0.51; 1.35] |
| ≥ 2.69 | 109 (21.25) | 0.90 [0.69; 1.18] | 77 (20.37) | 0.95 [0.69; 1.31] | 32 (23.70) | 0.81 [0.50; 1.31] |
| P-trend |  | 0.68 |  | 1.00 |  | 0.43 |
| Average daily UV dose during spring and summer in country of residence at inclusion (in kJ/m²) |  |  |  |  |  |  |
| < 2.36 | 107 (20.86) | Reference | 78 (20.63) | Reference | 29 (21.48) | Reference |
| [2.36 ; 2.48[ | 150 (29.24) | 1.08 [0.84; 1.39] | 109 (28.84) | 1.08 [0.81; 1.44] | 41 (30.37) | 1.09 [0.67; 1.75] |
| [2.48 ; 2.70[ | 136 (26.51) | 1.07 [0.83; 1.38] | 101 (26.72) | 1.09 [0.81; 1.47] | 35 (25.93) | 1.01 [0.62; 1.65] |
| ≥ 2.70 | 120 (23.39) | 0.91 [0.70; 1.19] | 90 (23.81) | 0.94 [0.70; 1.28] | 30 (22.22) | 0.83 [0.50; 1.38] |
| P-trend |  | 0.38 |  | 0.66 |  | 0.34 |

*For cases of CLL or NHL, totals do not add up because the category of missing values for each factor was not included in the table. There were 14 (2.73%) for skin sensitivity to sunlight ; 14 (2.73%) for number of nevi ; 12 (2.34%) for skin complexion; 11 (2.14%) for hair color ; 18 (3.51%) for number of freckles ; 40 (7.80%) for the average UV dose during spring and summer in location of residence at birth and no cases with missing values for the average UV dose during spring and summer in location of residence at inclusion.

Adjusted for age (as the time-scale), stratified by birth generation (< 1930, [1930 ; 1935[, [1935 ; 1940[, [1940 ; 1945[, ≥ 1945)
Abreviations: Confidence Interval (CI) ; Hazard-Ratio (HR) ; ultraviolet (UV) ; kilojoules by square meter (kJ/m²)
